# Effects of face coverings on people and interactions in mental health settings: scoping review

**DOI:** 10.1192/bjo.2025.10917

**Published:** 2025-12-12

**Authors:** Paul Van Houtte, Félix Lamarche, Susanna Every-Palmer

**Affiliations:** Mental Health, Addiction and Intellectual Disability Service, Health New Zealand/Te Whatu Ora Capital, Coast and Hutt Valley, Wellington, New Zealand; Department of Psychological Medicine, University of Otago, Wellington, New Zealand

**Keywords:** Mental health, psychiatry, masks, face covering, scoping review, autism spectrum disorder

## Abstract

**Background:**

Early in the SARS–CoV-2 pandemic, most jurisdictions implemented mandatory face covering policies across healthcare settings. This intervention, which lasted multiple years, was unprecedented in psychiatry. Masks may affect the delivery of mental healthcare, given its reliance on nuanced communication and establishing a therapeutic alliance.

**Aims:**

This scoping review aimed to provide an overview of the current literature concerning the impact of face masks in mental health settings beyond infection control and identify research gaps to guide future research and policy.

**Method:**

Systematic searches were completed in the MEDLINE, Embase, PsycINFO, Scopus and CINAHL databases on 14 August 2024. Articles were eligible if they described peer-reviewed empirical studies involving people with mental disorders or mental health clinicians that reported on impacts of face coverings.

**Results:**

Twenty-eight studies were selected for inclusion, involving 5385 participants. There was considerable heterogeneity among studies. Negative effects of face masks were reported in 26 studies in at least one domain. Themes from the survey-based literature included face masks negatively affecting communication, the therapeutic relationship and overall assessment quality. Experimental studies using emotion recognition tasks showed that people with mental disorders were disadvantaged by masks when interpreting emotions from facial expressions. The most commonly studied population was people with autism spectrum disorder. Children and people with severe or acute mental illness were underrepresented. Only two studies expressly recruited psychiatrists.

**Conclusions:**

Policy makers should be aware of adverse impacts of mask-wearing in mental health settings and consider these in evolving risk–benefit analyses. Further research is needed to establish the extent of impacts on population subgroups.

Face coverings have been used in various cultures and healthcare settings historically. Mask use became widespread during the SARS-CoV-2 pandemic, with many jurisdictions implementing broad mask mandates across healthcare settings in an attempt to reduce viral transmission. For several years, many health departments across the world continued to require or recommend regular use of face coverings in healthcare interactions, including in community and in-patient mental health settings. As of 2025, mask mandates have largely been discontinued. However, some public health advocates continue to recommend that the mandatory wearing of face coverings is incorporated into routine medical practice.^1–3^ This intervention may have significant implications for the delivery of psychiatric care owing to its reliance on nuanced communication.^4^

Face masks affect both verbal and non-verbal communication. Research has demonstrated that masks affect speech intelligibility,^5^ remove the ability to use supportive lip-reading cues^6^ and affect other elements of communication such as emotion recognition by obstructing facial expression.^7,8^ The magnitude of these impacts varies widely according to factors such as mask type, distance, background noise and listener characteristics.^6,9^ In addition to the impact on communication, some studies have found that face coverings can affect feelings of connection and engagement, particularly in medical settings.^10–12^

These features may act as barriers to mental health treatment. Mental health clinicians rely on clear verbal and non-verbal communication to build rapport and establish therapeutic alliances with patients.^13,14^ Visualisation of a client’s face is also needed for mental state assessments, such as appraisal of affect, which influences diagnostic impressions and clinical decision-making.^15,16^ Mental health patients present with a broad range of language and social communication difficulties, cognitive processing deficits or mentalising impairments.^17–21^ As such, we hypothesised that mental health patients and healthcare workers would be particularly vulnerable to the effects of face coverings on clinical interactions.

At the juncture of easing mandatory restrictions and future planning, this scoping review aimed to synthesise and critically examine the existing literature regarding masks in mental health settings. We also aimed to identify any research gaps and offer suggestions for future research.

## Method

This review followed the Preferred Reporting Items for Systematic Reviews and Meta-Analyses for Scoping Reviews (PRISMA-ScR) guidelines.^22^ A protocol was published *a priori* on 18 December 2023 (https://hdl.handle.net/10523/16481).

### Search strategy

A pilot search was developed based on the Population, Concept, and Context framework.^23^ The population was defined as all mental health patients and providers, to reflect the broad range of patients and clinician roles involved in psychiatric care. The concept in question was the impact of face coverings on the delivery of care and quality of interactions, as well as the impact on population members. The context was defined as any setting in which mental healthcare could be delivered, including psychiatric and psychotherapy settings, across in-patient, out-patient and community environments. The reviewers followed a recognised three-step search strategy as recommended by JBI.^23^

Initial pilot searches were conducted in MEDLINE and PsycINFO with librarian support. A sample set of references was established from pilot searches, which were used for data mining to obtain further key terms and subject headings. The search strategy was an iterative process which evolved as more data were obtained. A systematic search was conducted with the final search strategy on 14 August 2024 using the Embase, MEDLINE, PsycINFO, Scopus and CINAHL databases. Search terms included all of the most prevalent mental health disorders, mental health practitioner roles and psychotherapy modalities, such as ‘schizophrenia’, ‘borderline personality disorder’, ‘psychiatrist’, ‘psychologist’ and ‘cognitive behavioural therapy’, as well as general diagnostic categories such as ‘affective disorder’ and ‘dementia’, alongside appropriate Medical Subject Headings. Search terms for face coverings included ‘face cover*’, ‘face mask*’ and ‘N95’ among others.

We expected that by using broad search terms such as ‘mental health’ and ‘therapist*’, as well as pairing strategies such as ‘mental adj3’ with ‘patient*’, ‘client*’ or ‘consumer*’, we would capture rarer populations of interest for which we could not include specific search terms. Non-specific healthcare terms such as ‘doctor’, ‘nurse’ or ‘patient’ were not included individually, as our pilot searches suggested that these would expand the results beyond our capacity to screen, with a low chance of yielding further relevant results. We used established search string methodologies with combinations of mental health- ‘AND’ mask-related terms in the ‘Title, Abstract, Keyword’ sections of all databases to obtain search results.

An example search strategy, used for MEDLINE, is provided in Supplementary File 1 available at https://doi.org/10.1192/bjo.2025.10917. The complete search strategy for all databases is available in Supplementary File 2.

### Study inclusion and exclusion criteria

Our review considered all peer-reviewed empirical studies using quantitative, qualitative or mixed research designs, concerning the effects of face coverings on (a) mental health service providers, (b) mental health patients or people with formal psychiatric diagnoses according to the DSM-5 or ICD-11, (c) interactions between these parties or (d) the delivery of mental healthcare in any environment. Face coverings included any barrier obstructing the lower half of the face, for example, surgical, cloth or N-95 masks. Research needed to be published in full text in either English, French or German to be included. To enhance objectivity, the inclusion criteria were amended from the protocol to omit conceptual and review articles such as opinion pieces, as well as grey literature. Case studies were included in the initial eligibility criteria but later excluded in light of sufficient eligible empirical studies.

Studies were excluded if they concerned the general effects of the pandemic on the population of interest without specifically examining the effects of face coverings. We excluded research relating to the spread of infection, adherence rates or increasing mask-wearing tolerance in populations with mental disorders, as well as effects of face coverings on the mental well-being of the general population, e.g. community anxiety or distress. Studies looking at religious face coverings or full personal protective equipment were also excluded.

### Screening, study selection and data extraction

Two reviewers (P.V.H. and F.L.) independently screened titles and abstracts of search results using Rayyan software (2024 for macOS; Rayyan, Massachusetts, USA; https://www.rayyan.ai/) to identify potentially relevant studies according to the above eligibility criteria. Uncertainties were resolved through consensus after consulting a third reviewer (S.E.-P.).

Relevant sources of evidence were charted in a table which was used for data extraction. Two reviewers (P.V.H. and F.L.) independently extracted data from each study and summarised key findings pertaining to the research question in an iterative data-charting process. Data were extracted for country of origin, year of publication, study aim, number of participants, participant variables such as patient diagnosis, clinician role, setting, study methodology and key findings. Disagreements were discussed and resolved by consensus in consultation with a third reviewer (S.E.-P.). Four study authors were contacted for further clarification. Reviewers also examined each study for strengths and limitations, although this was not done systematically owing to the heterogeneity of study methodologies.

## Results

From 7467 initial results, 4125 records were identified as duplicates and removed. Titles and abstracts of the remaining 3342 were screened. Twenty-eight studies were eligible for inclusion.

**Fig. 1:**
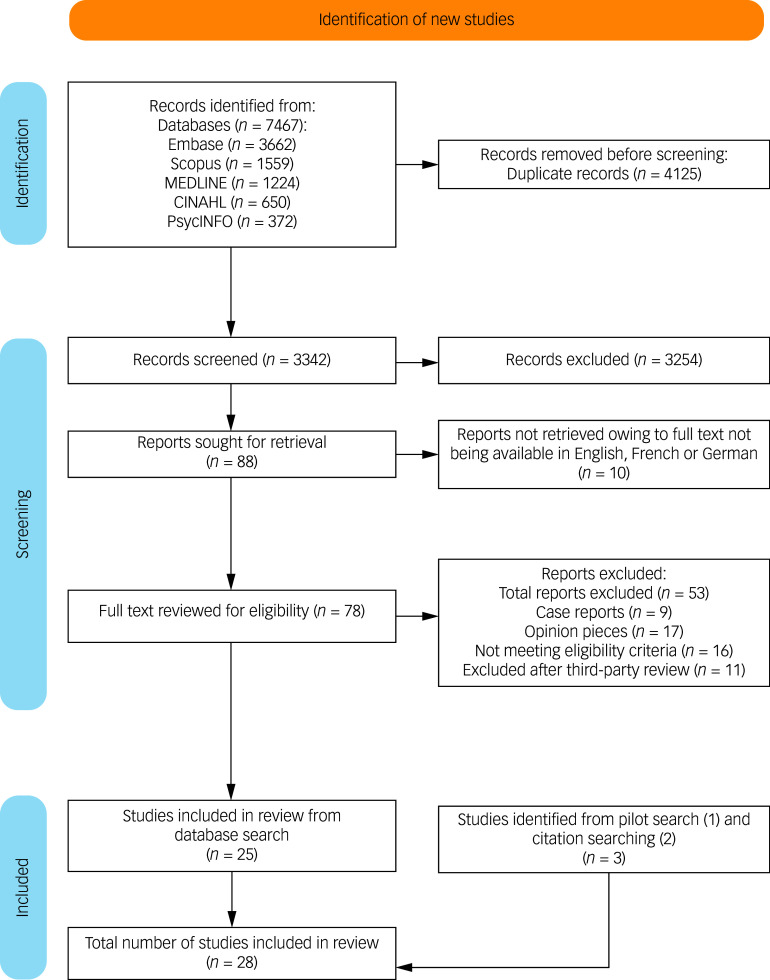
PRISMA flow chart.

A PRISMA flow diagram outlining the screening and selection process is presented in Fig. 1.^24^

### Characteristics of sources of evidence

Of the 28 studies, 16 used surveys or interview-based designs, 11 were experimental, including one randomised controlled trial (RCT), and there were two retrospective observational studies. Two contained both survey and experimental elements. Significant heterogeneity existed among studies. The majority examined the impacts of face coverings as a primary focus, with some exploring general pandemic effects with masks emerging as a topic.

Twenty studies recruited mental health patients, 11 studies recruited clinicians and three studies included both. One study featured caregivers of children with a mental disorder. The total number of participants was 5385. The majority of studies were conducted in out-patient or community settings. Six studies were based in Asia and 19 in Europe, with one based in both continents; three were North American and one South American. Although no date limit was set on searches, only one study set before the COVID-19 pandemic was identified.^25^

Most survey-based studies specified that both clinicians and patients were wearing masks during clinical encounters, two specified that only clinicians were masked, and in five studies this was unclear. The context, methodology and key findings of all included studies are summarised in Supplementary Table 1.

### Participant characteristics (patients)

Of 20 studies involving patients, eight concerned people with autism spectrum disorder (ASD). Six studies recruited mixed groups of patients with various psychiatric diagnoses, mostly mood, anxiety or somatoform disorders. The only study to specifically recruit participants with psychotic disorders was that of Escelsior et al.^26^ People with psychotic disorders were included in other studies but only in small numbers, with their effects not separated from those of other participants. There was a single study for each of the following diagnoses: major depressive disorder, anxiety disorders, borderline personality disorder, and attention-deficit hyperactivity disorder (ADHD). There were two studies on older adults with cognitive disorders. Only three studies considered children.

Studies recruiting patients comprised eight survey- or interview-based studies, nine studies using emotion or facial-recognition tasks, two observational *s*tudies exploring impacts on cognitive assessments and one RCT.

### Participant characteristics (clinicians)

In the 11 studies involving mental health service providers, the most common clinician role was psychotherapy, with seven studies recruiting psychotherapists. Two studies specifically recruited psychiatrists. Two studies included trainee clinician perspectives. Other clinician populations included psychiatric nurses, pharmacists and employees at a mental health trust. All 11 studies exploring clinicians’ perspectives used survey- or interview-based designs; one also contained an emotion recognition task.

### Findings

Of 28 studies, 26 reported that face masks had negative effects in at least one of several often overlapping domains. These included effects on verbal and non-verbal communication, emotion recognition or facial recognition; effects on interpersonal relationships such as the therapeutic alliance or connectedness; effects on morale or well-being; reduced diagnostic accuracy; impairment of quality of care in general; and symptom exacerbation. Seven studies noted some positive effects of masks, including increased feelings of safety and confidence for some patients.

### Communication

Thirteen studies reported on the effects of face masks on communication. All used either surveys or semi-structured interviews to gather data. Most of these studies explored general effects of masks on clinical processes, with effects on communication being a common topic that emerged. Of the 13 studies, 11 reported negative effects of masks on communication. These included negative effects on verbal communication, non-verbal communication or both, causing difficulties with expression and comprehension, impairing the gathering of clinical information, and at times leading to misinterpretation or misunderstandings.

All eight studies that considered communication from clinicians’ perspectives reported negative impacts of face coverings, and, where specified, this was reported by the majority of participants. Erschens et al^27^ and Kuczyk et al^28^ found that clinicians were more likely than patients to report that masks impaired communication.

Six of eight studies canvassing patient perspectives on communication reported negative impacts of masks. One Japanese study^29^ reported no difficulties in communication with masks, although this study contained only two closed questions from which generalisations were drawn. Kuczyk et al^28^ found that for people receiving psychotherapy, the communication barriers were not as significant as they had initially anticipated.

### Emotion recognition

Nine studies explored the effects of masks on emotion recognition by testing participants’ abilities to accurately interpret emotions from images of masked faces. All nine studies found that face masks compromised emotion recognition for all populations studied, supporting existing research in the general population.^8^ Eight of these studies assessed patient populations, and one assessed psychotherapists. The patient populations studied included people with autism (five studies), major depressive disorder (two studies), bipolar disorder, schizophrenia and borderline personality disorder (one study each). Results varied as to which emotions were more difficult to identify when masks were present.^30–32^

Seven studies compared people with mental disorders against control groups, all finding that people with mental health diagnoses significantly underperformed compared with controls in appraising both masked and unmasked faces, with the possible exception of individuals with bipolar disorder. Six studies sought to identify whether masks would ‘disproportionately’ affect the study population. Three of six found that those with mental health diagnoses were disproportionately affected by masks, i.e. the drop in performance was greater than that for controls; this was found for patients with depression, schizophrenia and ASD.^26,31,33^ The remaining three studies found equivalent reductions in emotion recognition accuracy for patient groups and controls, although patients had lower baselines; this was found for ASD and borderline personality disorder.^34–36^

The study of Escelsior et al^26^ was the only one to compare different mental disorders, finding that the detrimental effect of masks on emotion recognition appeared particularly pronounced for patients with schizophrenia and major depressive disorder in identifying low intensity positive emotions. Gehdu et al^35^ suggested that alexithymia was a stronger predictor of emotion recognition difficulties for masked faces than a diagnosis of autism.

Difficulties with emotion recognition were also reported in eight survey- or interview-based studies. A combination of clinicians, caregivers and patient populations reported difficulties interpreting facial expressions or expressing emotion, with effects on communication, interpersonal synchronisation and clinical assessment.

### Face recognition

Two experimental studies explored facial recognition with masks.^33,37^ Both studied people with ASD and found that they were disproportionately affected in their ability to recognise or learn masked faces compared with controls.

### Relationships

Twelve survey- or interview-based studies reported negative impacts of face coverings on interpersonal relationships or interactions between clinicians and patients. Nine of these explored clinician perspectives, describing general impacts on relationships or more specific effects regarding difficulties establishing a therapeutic alliance or rapport, reduced relational depth, reduced interpersonal synchrony and reduced connectedness with colleagues.

Patient perspectives were represented in four studies, with findings including masks hindering the therapeutic relationship with staff and other social interactions.

Two studies reported that masks were perceived as more of a barrier to the therapeutic relationship in encounters between unfamiliar people, compared with those with already established relationships.^38,39^ One experimental study^34^ found that people with borderline personality disorder perceived faces as less trustworthy than controls, and that the perception of trustworthiness was reduced equally by masks for both groups.

### Self-perception or clinician well-being

Five survey- or interview-based studies highlighted negative impacts of face masks on clinician self-perception or well-being.^27,30,40–42^ Descriptions included feelings of alienation or anonymisation, self-doubt, a reduction in morale or job satisfaction, burnout and compromised autonomy. Some studies also reported masks being physically uncomfortable and distracting.

### Diagnostic accuracy

Two studies looked at the effects of masks on some commonly used cognitive tests and screening tools for adults with or at risk of dementia. Okyar Bas et al^43^ found that masks reduced performance in screening tests, which may lead to overdiagnosis of cognitive impairments if standard diagnostic scoring thresholds are used. Thomas and Tranel^44^ found a drop in performance for older adults when masks were worn during verbally mediated but not visually mediated tests.

Four survey-based studies described biases and difficulties in the collection of clinical signs due to masks through reduced verbal and visual cues.^38,41,45,46^

### Symptom exacerbation

The single RCT included in this review, the study by Zhang et al,^47^ found that patients with diagnoses of anxiety disorders who were assigned to wear surgical or N95 masks for multiple weeks in the community experienced a significant increase in anxiety symptoms compared with the unmasked control group; this was especially pronounced for those wearing N95 respirator masks.

Three studies reported that some other symptoms were exacerbated by masks, including sensory difficulties,^48^ panic attacks^40^ and false beliefs such as persecutory ideation.^45^

### Overall quality of care

Several studies from clinician perspectives reported deteriorations in overall quality of care when masks were worn. This was described in general terms; for example, masks caused a deterioration in clinical practice quality,^45^ reduced the ability to perform one’s role or deliver care,^49,50^ and reduced clinicians’ confidence in their ability to be effective.^30^ This overall impact was most explicitly explored by Donde et al,^45^ who found that 94% of psychiatrists and trainees reported a deterioration in the overall quality of consultations. Erschens et al^40^ reported that the majority of clinicians but only a minority of patients considered face masks to hinder individual therapy. Two studies reported that despite some obstructive elements of masks in psychotherapy, most participants felt that the overall efficacy of assessment was preserved.^28,38^

### Positive impacts

Seven studies noted examples of positive effects of masks in interactions for some people with mental disorders. These included enhancing self-confidence,^28,41^ acting as a protective barrier to hide emotions,^40,48^ less pressure to ‘camouflage’ facial expression for people with ASD,^48^ creating a sense of solidarity between in-patients against masks as a ‘common evil’^40^ and reduced fear of infection;^29,48^ masks were also seen as enabling in-person therapy to occur during the pandemic.^38^

## Discussion

The negative impacts of masks on some specific populations, such as children and those with hearing impairments, have been considered in various reviews;^9,51,52^ however this scoping review appears to be the first to methodically examine the literature concerning face masks in relation to psychiatry. Our findings suggest that there are specific difficulties associated with mask use in mental health settings, and that more high-quality research is required.

The 28 studies included in this review explored a range of interrelated and overlapping topics related to the research question. For example, three of the main reported impacts were on emotion recognition, communication and the therapeutic relationship, each of which affects the others mutually.

The importance of the human face in communication and forming relationships has been extensively researched. The fields of social and cognitive neuroscience may provide a framework for understanding how face masks affect interactions in mental health contexts. Speech perception is a multisensory integration process, involving complementary verbal and non-verbal components.^51^ In the absence of lip reading cues, neural processing of auditory speech is slower.^51^ Other research supports a theory of holistic face processing^52^ and involvement of the mirror neuron system in face-to-face interactions.^53,54^ This raises questions around how interpersonal relations are affected if 60–70% of the face is covered by a mask.^12,55,56^ The mirror neuron system is thought to be involved in social cognition deficits in some psychiatric disorders such as schizophrenia,^57^ which has been conceptualised as a disorder of communication.^58^ Functional magnetic resonance imaging studies in healthy participants have demonstrated disruptive effects on brain connectivity when people interact with masked faces^7^ and wear masks themselves,^59^ including effects on the salience network, an area involved in communication, social behaviour, self-awareness and detection of environmental stimuli.^60^

Most studies included in this review were either survey-based studies exploring perspectives of patients and clinicians or experimental studies exploring the effects of masks on facial emotion recognition. A main finding from perspective-based studies was that patients and clinicians frequently reported that masks negatively affected verbal and/or non-verbal communication in mental health settings. All eight studies examining communication from clinicians’ perspectives found that clinicians considered masks to impair communication. This was consistent with the findings of a systematic review of mask use in general medicine,^9^ in which face masks impaired speech perception in normal-hearing and hearing-impaired populations. Communication plays a key part in the ‘Swiss cheese’ model of adverse events in healthcare,^61^ in which miscommunication can result in compromised care, misdiagnoses, medication errors and poor patient outcomes.^9^ It is well recognised that psychiatry is a specialty with multiple challenges regarding clinical communication,^62^ and our findings suggest that this is compromised by mask use.

Although mask-related concealment of facial expressions was often viewed as a communication issue, it also hindered the assessment of affect – an important part of the mental state exam. This concern, explored in one of two studies of psychiatrists,^45^ may be particularly salient in acute psychiatric settings, where misinterpreting clinical signs in assessments may have significant consequences.

Another frequently reported perspective was that masks negatively affected therapeutic relationships. The studies that examined this in the most depth focused on psychotherapy.^38,40,41^ A strong therapeutic alliance has been well established as a predictor of positive outcomes in psychotherapy,^63^ and a body of evidence supports the therapeutic alliance as central to psychiatric care.^14,64^ However, significant differences between psychotherapy and psychiatric settings limit the generalisability of psychotherapy-based findings.^65^ In acute psychiatric care, alliance formation is shaped by additional factors such as symptom severity, agitation, involuntary treatment, perceived coercion, multidisciplinary care and poor continuity, which may be further disrupted by face coverings.^64^ In our review, the two studies exploring psychiatrists’ views found that 62% and 47% perceived face coverings to negatively affect the therapeutic alliance.^45,66^

Several studies noted that some perceived impacts of masks could be influenced by personal beliefs. Masks can carry various subjective meanings, including protection from infection, symbolising fear, social responsibility, social conformity, barriers to connection, opportunities for self-concealment or infringements on autonomy with mandates.^67,68^ These meanings may change over time and are influenced by individual health, public health messaging and policies, and sociocultural values.^69,70^

It has been well established that masks reduce emotion recognition abilities among the general population,^8^ and this was consistent with the findings of our review. Across all studied populations, whether patients, clinicians or controls, masks reduced the accuracy of emotion recognition using standardised tests. Pre-pandemic research has shown that people with ASD and schizophrenia already experience emotion recognition deficits.^17,71^ Our review also supports these findings, as participants with mental disorders performed on average worse than controls on emotion recognition tasks in all studies.

Most emotion recognition studies focused on ASD, possibly because individuals with autism are believed to rely more on the mouth region than the eyes to infer emotion.^72^ Gehdu et al^35^ found that alexithymia predicted emotion recognition difficulties more strongly than an ASD diagnosis – a notable finding given the prevalence of alexithymia in other disorders.^73^

The patient populations found to be ‘disproportionately’ affected by masks included people with major depressive disorder (two studies) and schizophrenia (one study), with mixed findings for people with ASD. These patient groups may represent some of those most severely affected by the use of masks. Even proportionate reductions in emotion recognition are significant when starting from a lower baseline, as they may lead to greater functional impairment in already disadvantaged patient groups.^35^

### Limitations

This scoping review captures the state of research relating to face coverings in mental health contexts as of August 2024. Since the COVID-19 pandemic, this area of research has evolved rapidly, and new research may have been published since our search was conducted. The majority of the included studies were conducted early in the COVID-19 pandemic. Public and personal attitudes towards masks have shifted as the pandemic has eased, and it is possible that some of the perspectives represented in these studies would be different if the studies were repeated. Given the politicised and often polarised attitudes towards masks, researcher bias is of particular concern.^74^ Sampling biases may have been present in the perspective studies, as participants who choose to participate in surveys or actively comment in open-text questions may have stronger views. It is therefore possible that neutral perspectives are underrepresented. Conversely, social desirability biases may lead to participants underreporting adverse impacts owing to the perceived safety of masks or other cultural factors. Patients who consent to participate in studies may be higher-functioning or more trusting. Patient perspectives included those from community members with ASD or previous diagnosis of borderline personality disorder, out-patients with ADHD or anxiety, and in-patients receiving psychotherapy. Patients with severe mental disturbances such as acute psychosis, catatonia, delirium, advanced dementia or acute suicidality, as well as children, were generally excluded or not represented in studies examining patient perspectives. The only study looking specifically at people with psychosis explored emotion recognition deficits and not perspectives on masks.

There was considerable heterogeneity in study methodologies and participants. The preponderance of papers studying the effects of masks in a psychotherapy context hampered generalisability to psychiatric settings, in which effects may be more pronounced. Aside from the single RCT, experimental studies were performed in controlled settings using static images, and their outcomes may have differed significantly from real-world situations. In some interview-based studies, prevalence of reported impacts could not be determined. Closed questioning or broad interview topics may have led to omission or underreporting of specific impacts.

Importantly, data were lacking on the contribution of masks to the presence or absence of demonstrable adverse patient outcomes, such as increased use of coercion, reportable events, increased length of stay, misdiagnosis, suicide, aggression or therapy drop-out. The numerous disruptive elements from the pandemic and infection control strategies introduced confounding variables which may pose a barrier to attributing causality from the effects of face coverings to such outcomes. As a formal critical appraisal of the sources of evidence was outside of the scope of this review, studies were not individually assessed for quality or biases using standardised assessment tools.

### Recommendations for future research

Future research should prioritise those with severe mental illness and those less able to advocate for themselves, such as the patient populations mentioned above. Although challenges exist with respect to accessing the views of severely mentally unwell patients, people can usually indicate their preferences, and caregiver perspectives, as shown by Tamon et al,^75^ should also be considered. More studies involving psychiatrists are also warranted. Case reports excluded from our review indicated additional at risk groups, including people with post-traumatic stress disorder,^76,77^ suicidal ideation^78,79^ and tardive dyskinesia.^80^ Qualitative and quantitative studies focusing on face coverings could explore emerging themes such as impaired diagnostic accuracy; feelings of connectedness, well-being and autonomy; psychodynamic perspectives; and specific clinical outcomes. We encourage use of open-field text feedback areas in surveys to capture overlooked impacts.

Further RCTs are feasible, as shown by Zhang et al;^47^ however, ethical concerns about compromising care may limit trials in clinical practice outside public health crises. Studies such as McCabe’s trial to test communication training for psychiatrists could be adapted to test the impacts of masks in scenarios with consenting patients or healthy participants,^81^ using tools such as the Scale to Assess Therapeutic Relationship.^82^ Functional magnetic resonance imaging studies could also be undertaken to examine whether mask-related neural connectivity effects are more pronounced in psychiatric populations. We identified a substantial body of literature on the effects of masks on emotion recognition in individuals with ASD, which may justify a systematic review. Future research should also establish whether or not the adverse impacts of masks on psychiatric populations can be feasibly and meaningfully reduced by mitigation strategies, as current evidence for these is lacking.^83,84^

### Implications for practice and policy

Our findings demonstrate evidence of a range of negative effects associated with face masks in mental health settings. Our review suggests that face coverings negatively affect communication, therapeutic relationships and emotion recognition, with implications for clinical care. Policy makers and clinicians need to balance potential harms against potential benefits, and, given the conflicting evidence and overall low quality of studies on mask efficacy, further high-quality research in multiple settings is necessary to support evidence-based decision-making on future mask use.^85–90^ Further research is also needed to establish the extent of impacts on specific vulnerable patient populations and to follow-up on other themes from this review, such as negative effects on clinician mental well-being.

Future policy on mask use in mental health settings must incorporate these considerations into evolving risk–benefit analyses.

## Supporting information

Van Houtte et al. supplementary material 1 Van Houtte et al. supplementary material

Van Houtte et al. supplementary material 2 Van Houtte et al. supplementary material

Van Houtte et al. supplementary material 3 Van Houtte et al. supplementary material

## Data Availability

Data sharing is not applicable to this article as no new data were generated or analysed in this study.

## Supplementary material

The supplementary material is available online at https://doi.org/10.1192/bjo.2025.10917
