# Effects of face coverings on people and interactions in mental health settings: scoping review – ERRATUM

**DOI:** 10.1192/bjo.2025.10962

**Published:** 2026-01-07

**Authors:** Paul Van Houtte, Félix Lamarche, Susanna Every-Palmer

**Keywords:** Mental health, psychiatry, masks, face covering, scoping review, autism spectrum disorder, erratum

In the original publication of this article, supplementary material 3 contained referencing errors.

This has since been updated to be correct. The Publisher apologises for this error.

## Supporting information

Van Houtte et al. supplementary material Van Houtte et al. supplementary material
