# Supplementary figures and images for: Effects of face coverings on people and interactions in mental health settings: scoping review
Source: BJPsych Open. 2025 Dec 12;12(1):e11. doi: 10.1192/bjo.2025.10917 (PMC12724123; doi:10.1192/bjo.2025.10917)

**Supplementary File 1.** Example search strategy, used in Medline on August 14 2024


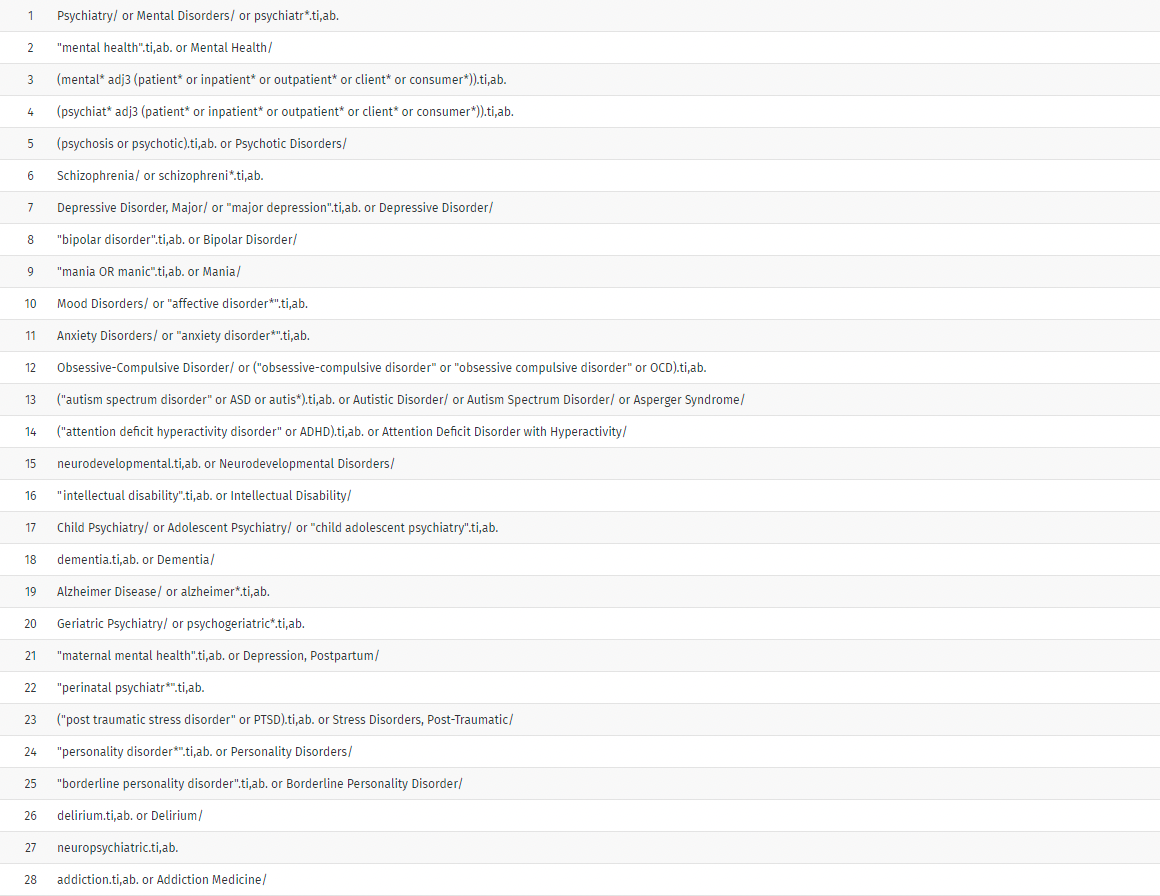


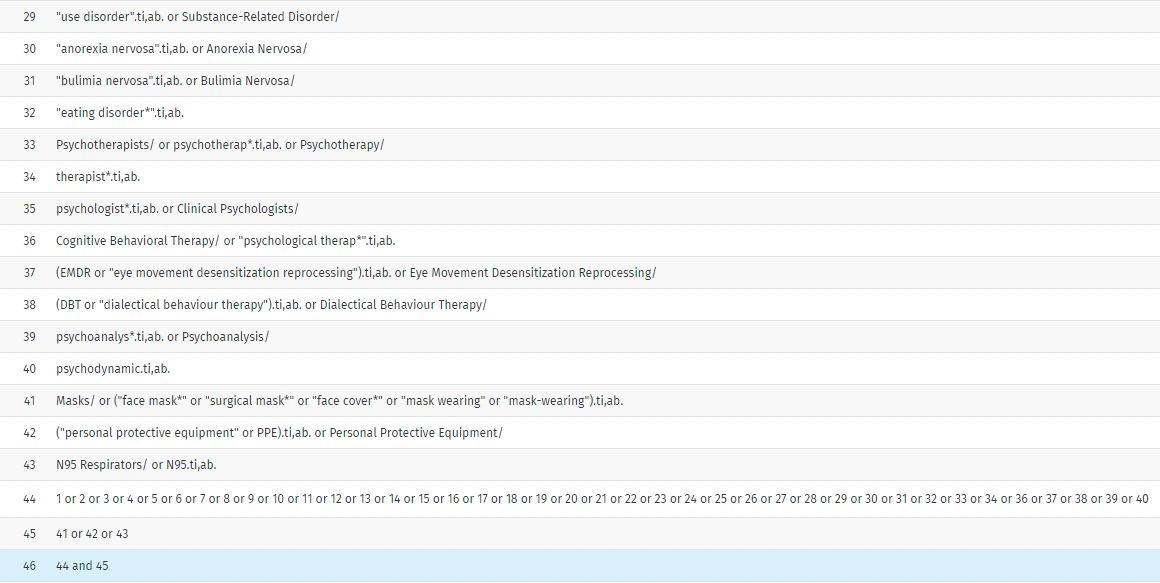

Supplement: Van Houtte et al. supplementary material 1 — Van Houtte et al. supplementary material [file S2056472425109174sup001.docx]
