# Supplementary material for: Effects of face coverings on people and interactions in mental health settings: scoping review
Source: BJPsych Open. 2025 Dec 12;12(1):e11. doi: 10.1192/bjo.2025.10917 (PMC12724123; doi:10.1192/bjo.2025.10917)
Supplement: Van Houtte et al. supplementary material 2 — Van Houtte et al. supplementary material [file S2056472425109174sup002.docx]

**Supplementary File 2.** Full search strategies for all databases

Embase

| 1 | Psychiatry/ or Mental Disorders/ or psychiatr*.ti,ab. | 526518 | Advanced |
| --- | --- | --- | --- |
| 2 | "mental health".ti,ab. or Mental Health/ | 385719 | Advanced |
| 3 | (mental* adj3 (patient* or inpatient* or outpatient* or client* or consumer*)).ti,ab. | 40109 | Advanced |
| 4 | (psychiat* adj3 (patient* or inpatient* or outpatient* or client* or consumer*)).ti,ab. | 62218 | Advanced |
| 5 | (psychosis or psychotic).ti,ab. or Psychotic Disorders/ | 131642 | Advanced |
| 6 | Schizophrenia/ or schizophreni*.ti,ab. | 238950 | Advanced |
| 7 | Depressive Disorder, Major/ or "major depression".ti,ab. or Depressive Disorder/ | 312952 | Advanced |
| 8 | "bipolar disorder".ti,ab. or Bipolar Disorder/ | 83516 | Advanced |
| 9 | "mania OR manic".ti,ab. or Mania/ | 21046 | Advanced |
| 10 | Mood Disorders/ or "affective disorder*".ti,ab. | 47456 | Advanced |
| 11 | Anxiety Disorders/ or "anxiety disorder*".ti,ab. | 97146 | Advanced |
| 12 | Obsessive-Compulsive Disorder/ or ("obsessive-compulsive disorder" or "obsessive compulsive disorder" or OCD).ti,ab. | 41340 | Advanced |
| 13 | ("autism spectrum disorder" or ASD or autis*).ti,ab. or Autistic Disorder/ or Autism Spectrum Disorder/ or Asperger Syndrome/ | 124977 | Advanced |
| 14 | ("attention deficit hyperactivity disorder" or ADHD).ti,ab. or Attention Deficit Disorder with Hyperactivity/ | 64523 | Advanced |
| 15 | neurodevelopmental.ti,ab. or Neurodevelopmental Disorders/ | 298226 | Advanced |
| 16 | "intellectual disability".ti,ab. or Intellectual Disability/ | 42197 | Advanced |
| 17 | Child Psychiatry/ or Adolescent Psychiatry/ or "child adolescent psychiatry".ti,ab. | 23671 | Advanced |
| 18 | dementia.ti,ab. or Dementia/ | 248922 | Advanced |
| 19 | Alzheimer Disease/ or alzheimer*.ti,ab. | 318749 | Advanced |
| 20 | Geriatric Psychiatry/ or psychogeriatric*.ti,ab. | 10845 | Advanced |
| 21 | "maternal mental health".ti,ab. or Depression, Postpartum/ | 10147 | Advanced |
| 22 | "perinatal psychiatr*".ti,ab. | 424 | Advanced |
| 23 | ("post traumatic stress disorder" or PTSD).ti,ab. or Stress Disorders, Post-Traumatic/ | 75862 | Advanced |
| 24 | "personality disorder*".ti,ab. or Personality Disorders/ | 42649 | Advanced |
| 25 | "borderline personality disorder".ti,ab. or Borderline Personality Disorder/ | 15300 | Advanced |
| 26 | delirium.ti,ab. or Delirium/ | 48170 | Advanced |
| 27 | neuropsychiatric.ti,ab. | 63094 | Advanced |
| 28 | addiction.ti,ab. or Addiction Medicine/ | 82330 | Advanced |
| 29 | "use disorder".ti,ab. or Substance-Related Disorder/ | 94539 | Advanced |
| 30 | "anorexia nervosa".ti,ab. or Anorexia Nervosa/ | 26356 | Advanced |
| 31 | "bulimia nervosa".ti,ab. or Bulimia Nervosa/ | 14917 | Advanced |
| 32 | "eating disorder*".ti,ab. | 34369 | Advanced |
| 33 | Psychotherapists/ or psychotherap*.ti,ab. or Psychotherapy/ | 128056 | Advanced |
| 34 | therapist*.ti,ab. | 78480 | Advanced |
| 35 | psychologist*.ti,ab. or Clinical Psychologists/ | 32288 | Advanced |
| 36 | Cognitive Behavioral Therapy/ or "psychological therap*".ti,ab. | 32946 | Advanced |
| 37 | (EMDR or "eye movement desensitization reprocessing").ti,ab. or Eye Movement Desensitization Reprocessing/ | 1561 | Advanced |
| 38 | (DBT or "dialectical behaviour therapy").ti,ab. or Dialectical Behaviour Therapy/ | 5920 | Advanced |
| 39 | psychoanalys*.ti,ab. or Psychoanalysis/ | 36329 | Advanced |
| 40 | psychodynamic.ti,ab. | 8951 | Advanced |
| 41 | Masks/ or ("face mask*" or "surgical mask*" or "face cover*" or "mask wearing" or "mask-wearing").ti,ab. | 21201 | Advanced |
| 42 | ("personal protective equipment" or PPE).ti,ab. or Personal Protective Equipment/ | 37801 | Advanced |
| 43 | N95 Respirators/ or N95.ti,ab. | 7447 | Advanced |
| 44 | 1 or 2 or 3 or 4 or 5 or 6 or 7 or 8 or 9 or 10 or 11 or 12 or 13 or 14 or 15 or 16 or 17 or 18 or 19 or 20 or 21 or 22 or 23 or 24 or 25 or 26 or 27 or 28 or 29 or 30 or 31 or 32 or 33 or 34 or 36 or 37 or 38 or 39 or 40 | 2341113 | Advanced |
| 45 | 41 or 42 or 43 | 62332 | Advanced |
| 46 | 44 and 45 | 3662 | Advanced |

**Scopus**

psychiatr* OR "mental health" OR ( mental* W/3 ( patient* OR inpatient* OR outpatient* OR client* OR consumer* ) ) OR ( psychiat* W/3 ( patient* OR inpatient* OR outpatient* OR client* OR consumer* ) ) OR psychosis OR psychotic OR schizophreni* OR "major depression" OR "bipolar disorder" OR mania OR manic OR "affective disorder*" OR "anxiety disorder*" OR "obsessive-compulsive disorder" OR "obsessive compulsive disorder" OR ocd OR “personality disorder*” OR "autism spectrum disorder" OR asd OR autis* OR "attention deficit hyperactivity disorder" OR adhd OR "neurodevelopmental disorder*" OR "intellectual disabilit*" OR "child adolescent psychiatr*" OR dementia OR alzheimer* OR psychogeriatric* OR delirium OR neuropsychiatric OR "maternal mental health" OR "perinatal psychiatr*" OR "post traumatic stress disorder" OR ptsd OR addiction OR “use disorder” OR anorexia OR bulimia OR "eating disorder*" OR psychotherap* OR therapist* OR psychologist* OR "psychological therap*" OR “cognitive behaviour* therap*” OR cbt OR “eye movement desensitization reprocessing therapy” OR emdr Or psychodynamic OR pychoanalys*

AND

"face mask*" OR "face cover*" OR "surgical mask*" OR "mask wearing" OR "mask-wearing" OR "personal protective equipment" OR ppe OR "N95 Respirators"

**Medline**

| 1 | Psychiatry/ or Mental Disorders/ or psychiatr*.ti,ab. | 416965 | Advanced |
| --- | --- | --- | --- |
| 2 | "mental health".ti,ab. or Mental Health/ | 253770 | Advanced |
| 3 | (mental* adj3 (patient* or inpatient* or outpatient* or client* or consumer*)).ti,ab. | 29687 | Advanced |
| 4 | (psychiat* adj3 (patient* or inpatient* or outpatient* or client* or consumer*)).ti,ab. | 44757 | Advanced |
| 5 | (psychosis or psychotic).ti,ab. or Psychotic Disorders/ | 96545 | Advanced |
| 6 | Schizophrenia/ or schizophreni*.ti,ab. | 164561 | Advanced |
| 7 | Depressive Disorder, Major/ or "major depression".ti,ab. or Depressive Disorder/ | 124941 | Advanced |
| 8 | "bipolar disorder".ti,ab. or Bipolar Disorder/ | 58167 | Advanced |
| 9 | "mania OR manic".ti,ab. or Mania/ | 650 | Advanced |
| 10 | Mood Disorders/ or "affective disorder*".ti,ab. | 31600 | Advanced |
| 11 | Anxiety Disorders/ or "anxiety disorder*".ti,ab. | 68883 | Advanced |
| 12 | Obsessive-Compulsive Disorder/ or ("obsessive-compulsive disorder" or "obsessive compulsive disorder" or OCD).ti,ab. | 24440 | Advanced |
| 13 | ("autism spectrum disorder" or ASD or autis*).ti,ab. or Autistic Disorder/ or Autism Spectrum Disorder/ or Asperger Syndrome/ | 84931 | Advanced |
| 14 | ("attention deficit hyperactivity disorder" or ADHD).ti,ab. or Attention Deficit Disorder with Hyperactivity/ | 50118 | Advanced |
| 15 | neurodevelopmental.ti,ab. or Neurodevelopmental Disorders/ | 46899 | Advanced |
| 16 | "intellectual disability".ti,ab. or Intellectual Disability/ | 72951 | Advanced |
| 17 | Child Psychiatry/ or Adolescent Psychiatry/ or "child adolescent psychiatry".ti,ab. | 7259 | Advanced |
| 18 | dementia.ti,ab. or Dementia/ | 159088 | Advanced |
| 19 | Alzheimer Disease/ or alzheimer*.ti,ab. | 211751 | Advanced |
| 20 | Geriatric Psychiatry/ or psychogeriatric*.ti,ab. | 3945 | Advanced |
| 21 | "maternal mental health".ti,ab. or Depression, Postpartum/ | 9767 | Advanced |
| 22 | "perinatal psychiatr*".ti,ab. | 188 | Advanced |
| 23 | ("post traumatic stress disorder" or PTSD).ti,ab. or Stress Disorders, Post-Traumatic/ | 57375 | Advanced |
| 24 | "personality disorder*".ti,ab. or Personality Disorders/ | 38196 | Advanced |
| 25 | "borderline personality disorder".ti,ab. or Borderline Personality Disorder/ | 10561 | Advanced |
| 26 | delirium.ti,ab. or Delirium/ | 24179 | Advanced |
| 27 | neuropsychiatric.ti,ab. | 43422 | Advanced |
| 28 | addiction.ti,ab. or Addiction Medicine/ | 57099 | Advanced |
| 29 | "use disorder".ti,ab. or Substance-Related Disorder/ | 130513 | Advanced |
| 30 | "anorexia nervosa".ti,ab. or Anorexia Nervosa/ | 19287 | Advanced |
| 31 | "bulimia nervosa".ti,ab. or Bulimia Nervosa/ | 7067 | Advanced |
| 32 | "eating disorder*".ti,ab. | 26491 | Advanced |
| 33 | Psychotherapists/ or psychotherap*.ti,ab. or Psychotherapy/ | 86594 | Advanced |
| 34 | therapist*.ti,ab. | 50602 | Advanced |
| 35 | psychologist*.ti,ab. or Clinical Psychologists/ | 20439 | Advanced |
| 36 | Cognitive Behavioral Therapy/ or "psychological therap*".ti,ab. | 34106 | Advanced |
| 37 | (EMDR or "eye movement desensitization reprocessing").ti,ab. or Eye Movement Desensitization Reprocessing/ | 964 | Advanced |
| 38 | (DBT or "dialectical behaviour therapy").ti,ab. or Dialectical Behaviour Therapy/ | 3978 | Advanced |
| 39 | psychoanalys*.ti,ab. or Psychoanalysis/ | 14091 | Advanced |
| 40 | psychodynamic.ti,ab. | 6018 | Advanced |
| 41 | Masks/ or ("face mask*" or "face cover*" or "surgical mask*" or "mask wearing" or "mask-wearing").ti,ab. | 14573 | Advanced |
| 42 | ("personal protective equipment" or PPE).ti,ab. or Personal Protective Equipment/ | 15353 | Advanced |
| 43 | N95 Respirators/ or N95.ti,ab. | 3308 | Advanced |
| 44 | 1 or 2 or 3 or 4 or 5 or 6 or 7 or 8 or 9 or 10 or 11 or 12 or 13 or 14 or 15 or 16 or 17 or 18 or 19 or 20 or 21 or 22 or 23 or 24 or 25 or 26 or 27 or 28 or 29 or 30 or 31 or 32 or 33 or 34 or 36 or 37 or 38 or 39 or 40 | 1733275 | Advanced |
| 45 | 41 or 42 or 43 | 30831 | Advanced |
| 46 | 44 and 45 | 1224 | Advanced |

**PsycInfo**

|  |  |  |  |  |
| --- | --- | --- | --- | --- |
| 1 | Psychiatry/ or Mental Disorders/ or psychiatr*.ti,ab. | 341506 | | Advanced |
| 2 | "mental health".ti,ab. or Mental Health/ | 263531 | | Advanced |
| 3 | (mental* adj3 (patient* or inpatient* or outpatient* or client* or consumer*)).ti,ab. | 24060 | | Advanced |
| 4 | (psychiat* adj3 (patient* or inpatient* or outpatient* or client* or consumer*)).ti,ab. | 46358 | | Advanced |
| 5 | (psychosis or psychotic).ti,ab. or Psychotic Disorders/ | 80498 | | Advanced |
| 6 | Schizophrenia/ or schizophreni*.ti,ab. | 137418 | | Advanced |
| 7 | Depressive Disorder, Major/ or "major depression".ti,ab. or Depressive Disorder/ | 40236 | | Advanced |
| 8 | "bipolar disorder".ti,ab. or Bipolar Disorder/ | 39296 | | Advanced |
| 9 | Psychotherapists/ or psychotherap*.ti,ab. or Psychotherapy/ | 138414 | | Advanced |
| 10 | therapist*.ti,ab. | 95586 | | Advanced |
| 11 | Cognitive Behavioral Therapy/ or "psychological therap*".ti,ab. | 3394 | | Advanced |
| 12 | mania.ti,ab. or Mania/ | 13203 | | Advanced |
| 13 | Mood Disorders/ or "affective disorder*".ti,ab. | 29675 | | Advanced |
| 14 | Anxiety Disorders/ or "anxiety disorder*".ti,ab. | 48064 | | Advanced |
| 15 | ("autism spectrum disorder" or ASD or autis*).ti,ab. or Autistic Disorder/ or Autism Spectrum Disorder/ or Asperger Syndrome/ | 69345 | | Advanced |
| 16 | ("attention deficit hyperactivity disorder" or ADHD).ti,ab. or Attention Deficit Disorder with Hyperactivity/ | 41074 | | Advanced |
| 17 | neurodevelopmental.ti,ab. or Neurodevelopmental Disorders/ | 18292 | | Advanced |
| 18 | dementia.ti,ab. or Dementia/ | 80979 | | Advanced |
| 19 | "intellectual disability".ti,ab. or Intellectual Disability/ | 48769 | | Advanced |
| 20 | Alzheimer Disease/ or alzheimer*.ti,ab. | 75850 | | Advanced |
| 21 | Obsessive-Compulsive Disorder/ or ("obsessive-compulsive disorder" or "obsessive compulsive disorder" or OCD).ti,ab. | 21486 | | Advanced |
| 22 | Geriatric Psychiatry/ or psychogeriatric*.ti,ab. | 3224 | | Advanced |
| 23 | Child Psychiatry/ or Adolescent Psychiatry/ or "child adolescent psychiatry".ti,ab. | 11124 | | Advanced |
| 24 | "maternal mental health".ti,ab. or Depression, Postpartum/ | 1379 | | Advanced |
| 25 | "perinatal psychiatr*".ti,ab. | 215 | | Advanced |
| 26 | ("post traumatic stress disorder" or PTSD).ti,ab. or Stress Disorders, Post-Traumatic/ | 45786 | | Advanced |
| 27 | delirium.ti,ab. or Delirium/ | 7955 | | Advanced |
| 28 | neuropsychiatric.ti,ab. | 23134 | | Advanced |
| 29 | addiction.ti,ab. or Addiction Medicine/ | 46618 | | Advanced |
| 30 | "anorexia nervosa".ti,ab. or Anorexia Nervosa/ | 16139 | | Advanced |
| 31 | "bulimia nervosa".ti,ab. or Bulimia Nervosa/ | 7201 | | Advanced |
| 32 | "eating disorder*".ti,ab. | 29738 | | Advanced |
| 33 | Masks/ or ("face mask*" or "face covering*" or "surgical mask*" or "mask wearing" or "mask-wearing").ti,ab. | 985 | | Advanced |
| 34 | ("personal protective equipment" or PPE).ti,ab. or Personal Protective Equipment/ | 1400 | | Advanced |
| 35 | N95 Respirators/ or N95.ti,ab. | 153 | | Advanced |
| 36 | 1 or 2 or 3 or 4 or 5 or 6 or 7 or 8 or 9 or 10 or 11 or 12 or 13 or 14 or 15 or 16 or 17 or 18 or 19 or 20 or 21 or 22 or 23 or 24 or 25 or 26 or 27 or 28 or 29 or 30 or 31 or 32 | 1211404 | | Advanced |
| 37 | 33 or 34 or 35 | 2176 | | Advanced |
| 38 | 36 and 37 | 372 | | Advanced |
| 39 | Psychiatry/ or Mental Disorders/ or psychiatr*.ti,ab. | 341506 | | Advanced |
| 40 | "mental health".ti,ab. or Mental Health/ | 263531 | | Advanced |
| 41 | (mental* adj3 (patient* or inpatient* or outpatient* or client* or consumer*)).ti,ab. | 24060 | | Advanced |
| 42 | (psychiat* adj3 (patient* or inpatient* or outpatient* or client* or consumer*)).ti,ab. | 46358 | | Advanced |
| 43 | (psychosis or psychotic).ti,ab. or Psychotic Disorders/ | 80498 | | Advanced |
| 44 | Schizophrenia/ or schizophreni*.ti,ab. | 137418 | | Advanced |
| 45 | Depressive Disorder, Major/ or "major depression".ti,ab. or Depressive Disorder/ | 40236 | | Advanced |
| 46 | "bipolar disorder".ti,ab. or Bipolar Disorder/ | 39296 | | Advanced |
| 47 | Psychotherapists/ or psychotherap*.ti,ab. or Psychotherapy/ | 138414 | | Advanced |
| 48 | therapist*.ti,ab. | 95586 | | Advanced |
| 49 | Cognitive Behavioral Therapy/ or "psychological therap*".ti,ab. | 3394 | | Advanced |
| 50 | mania.ti,ab. or Mania/ | 13203 | | Advanced |
| 51 | Mood Disorders/ or "affective disorder*".ti,ab. | 29675 | | Advanced |
| 52 | Anxiety Disorders/ or "anxiety disorder*".ti,ab. | 48064 | | Advanced |
| 53 | ("autism spectrum disorder" or ASD or autis*).ti,ab. or Autistic Disorder/ or Autism Spectrum Disorder/ or Asperger Syndrome/ | 69345 | | Advanced |
| 54 | ("attention deficit hyperactivity disorder" or ADHD).ti,ab. or Attention Deficit Disorder with Hyperactivity/ | 41074 | | Advanced |
| 55 | neurodevelopmental.ti,ab. or Neurodevelopmental Disorders/ | 18292 | | Advanced |
| 56 | dementia.ti,ab. or Dementia/ | 80979 | | Advanced |
| 57 | "intellectual disability".ti,ab. or Intellectual Disability/ | 48769 | | Advanced |
| 58 | Alzheimer Disease/ or alzheimer*.ti,ab. | 75850 | | Advanced |
| 59 | Obsessive-Compulsive Disorder/ or ("obsessive-compulsive disorder" or "obsessive compulsive disorder" or OCD).ti,ab. | 21486 | | Advanced |
| 60 | Geriatric Psychiatry/ or psychogeriatric*.ti,ab. | 3224 | | Advanced |
| 61 | Child Psychiatry/ or Adolescent Psychiatry/ or "child adolescent psychiatry".ti,ab. | 11124 | | Advanced |
| 62 | "maternal mental health".ti,ab. or Depression, Postpartum/ | 1379 | | Advanced |
| 63 | "perinatal psychiatr*".ti,ab. | 215 | | Advanced |
| 64 | ("post traumatic stress disorder" or PTSD).ti,ab. or Stress Disorders, Post-Traumatic/ | 45786 | | Advanced |
| 65 | delirium.ti,ab. or Delirium/ | 7955 | | Advanced |
| 66 | neuropsychiatric.ti,ab. | 23134 | | Advanced |
| 67 | addiction.ti,ab. or Addiction Medicine/ | 46618 | | Advanced |
| 68 | "anorexia nervosa".ti,ab. or Anorexia Nervosa/ | 16139 | | Advanced |
| 69 | "bulimia nervosa".ti,ab. or Bulimia Nervosa/ | 7201 | | Advanced |
| 70 | "eating disorder*".ti,ab. | 29738 | | Advanced |
| 71 | Masks/ or ("face mask*" or "face covering*" or "surgical mask*" or "mask wearing" or "mask-wearing").ti,ab. | 985 | | Advanced |
| 72 | ("personal protective equipment" or PPE).ti,ab. or Personal Protective Equipment/ | 1400 | | Advanced |
| 73 | N95 Respirators/ or N95.ti,ab. | 153 | | Advanced |
| 74 | 39 or 40 or 41 or 42 or 43 or 44 or 45 or 46 or 47 or 48 or 49 or 50 or 51 or 52 or 53 or 54 or 55 or 56 or 57 or 58 or 59 or 60 or 61 or 62 or 63 or 64 or 65 or 66 or 67 or 68 or 69 or 70 | 1211404 | | Advanced |
| 75 | 71 or 72 or 73 | 2176 | | Advanced |
| 76 | 74 and 75 | 372 | | Advanced |
|  | | |  |  |

**CINAHL**

| S23 | S21 AND S22 | Expanders - Apply equivalent subjects Search modes - Proximity |  | 647 | [Edit](javascript:__doPostBack('ctl00$ctl00$MainContentArea$MainContentArea$editControl$printHistory$HistoryRepeater$ctl00$linkEditSearch',''))S23 |
| --- | --- | --- | --- | --- | --- |
| S22 | S1 OR S2 OR S3 OR S4 OR S5 OR S6 OR S7 OR S8 OR S9 OR S10 OR S11 OR S12 OR S13 OR S14 OR S15 OR S16 OR S17 | Expanders - Apply equivalent subjects Search modes - Proximity |  | 1,001,369 | [Edit](javascript:__doPostBack('ctl00$ctl00$MainContentArea$MainContentArea$editControl$printHistory$HistoryRepeater$ctl01$linkEditSearch',''))S22 |
| S21 | S18 OR S19 OR S20 | Expanders - Apply equivalent subjects Search modes - Proximity |  | 9,152 | [Edit](javascript:__doPostBack('ctl00$ctl00$MainContentArea$MainContentArea$editControl$printHistory$HistoryRepeater$ctl02$linkEditSearch',''))S21 |
| S20 | (TI "n95 respirator*") OR (AB "N95 respirator*) OR (SU "n95 respirator*) OR (TI "n95 mask*") OR (AB "n95 mask*") OR (SU "n95 mask*") | Expanders - Apply equivalent subjects Search modes - Proximity |  | 387 | [Edit](javascript:__doPostBack('ctl00$ctl00$MainContentArea$MainContentArea$editControl$printHistory$HistoryRepeater$ctl03$linkEditSearch',''))S20 |
| S19 | (TI "personal protective equipment") OR (AB "personal protective equipment") OR (SU "personal protective equipment") OR (TI "respiratory protective device*") OR (AB "respiratory protective device*) OR (SU "respiratory protective device*") OR (TI "ppe") OR (AB "ppe") OR (SU "ppe") | Expanders - Apply equivalent subjects Search modes - Proximity |  | 6,939 | [Edit](javascript:__doPostBack('ctl00$ctl00$MainContentArea$MainContentArea$editControl$printHistory$HistoryRepeater$ctl04$linkEditSearch',''))S19 |
| S18 | (TI "face mask*") OR (AB "face mask*") OR (SU "face mask*") OR (TI "face covering") OR (AB "face covering") OR (SU "face covering") OR (TI "surgical mask*") OR (AB "surgical mask*) OR (SU "surgical mask*") OR (TI "mask wearing") OR (AB "mask wearing") OR (SU "mask wearing")OR (TI "mask-wearing") OR (AB "mask wearing") OR (SU "mask wearing") OR (TI "surgical mask*") or (AB "surgical mask*") OR (SU "surgical mask*) OR (TI "disposable mask*") OR (AB "disposable mask*" OR (SU "disposable mask*") OR (TI "medical mask*") OR (AB "medical mask*" OR (SU "medical mask*") | Expanders - Apply equivalent subjects Search modes - Proximity |  | 2,168 | [Edit](javascript:__doPostBack('ctl00$ctl00$MainContentArea$MainContentArea$editControl$printHistory$HistoryRepeater$ctl05$linkEditSearch',''))S18 |
| S17 | (TI "eye movement desensiti* and reprogramming") OR (AB "eye movement desensiti* and reprogramming") OR (SU "eye movement desensiti* and reprogramming") OR (TI "emdr") OR (AB "emdr") OR (SU "emdr") | Expanders - Apply equivalent subjects Search modes - Proximity |  | 562 | [Edit](javascript:__doPostBack('ctl00$ctl00$MainContentArea$MainContentArea$editControl$printHistory$HistoryRepeater$ctl06$linkEditSearch',''))S17 |
| S16 | (TI "Psychologist*") OR (AB "Psychologist*") OR (SU "psychologist*") OR (TI "psychoanalys*") OR (AB "psychoanalys*") OR (SU "psychoanalys*") OR (TI "psychotherap*") OR (AB "psychotherap*") OR (SU "psychotherap*") OR (AB "psychodynamic") OR (TI "psychodynamic") OR (SU "psychodynamic") | Expanders - Apply equivalent subjects Search modes - Proximity |  | 59,681 | [Edit](javascript:__doPostBack('ctl00$ctl00$MainContentArea$MainContentArea$editControl$printHistory$HistoryRepeater$ctl07$linkEditSearch',''))S16 |
| S15 | (TI "psychological therapy") OR (AB "psychological therapy") OR (SU "psychological therapy") | Expanders - Apply equivalent subjects Search modes - Proximity |  | 3,753 | [Edit](javascript:__doPostBack('ctl00$ctl00$MainContentArea$MainContentArea$editControl$printHistory$HistoryRepeater$ctl08$linkEditSearch',''))S15 |
| S14 | (TI "borderline personality disorder") OR (AB "borderline personality disorder") OR (SU "borderline personality disorder") OR (TI "Personality Disorders") OR (AB "Personality Disorders") OR (SU "Personality Disorders") | Expanders - Apply equivalent subjects Search modes - Proximity |  | 10,324 | [Edit](javascript:__doPostBack('ctl00$ctl00$MainContentArea$MainContentArea$editControl$printHistory$HistoryRepeater$ctl09$linkEditSearch',''))S14 |
| S13 | (TI "eating disorder*") OR (AB "eating disorder*") OR (SU "eating disorder*") OR (TI "anorexia nervosa") OR (AB "anorexia nervosa") or (SU "anorexia nervosa") OR (TI "bulimia nervosa") OR (AB "bulimia nervosa") OR (SU "bulimia nervosa") | Expanders - Apply equivalent subjects Search modes - Proximity |  | 23,355 | [Edit](javascript:__doPostBack('ctl00$ctl00$MainContentArea$MainContentArea$editControl$printHistory$HistoryRepeater$ctl10$linkEditSearch',''))S13 |
| S12 | (TI "addiction") OR (AB "addiction")(SU "addiction") OR (TI "substance use disorder") (AB "substance use disorder") OR (SU "substance use disorder") | Expanders - Apply equivalent subjects Search modes - Proximity |  | 10,042 | [Edit](javascript:__doPostBack('ctl00$ctl00$MainContentArea$MainContentArea$editControl$printHistory$HistoryRepeater$ctl11$linkEditSearch',''))S12 |
| S11 | (TI "maternal mental health") OR (AB "maternal mental health") OR (SU "maternal mental health") OR (TI "perinatal psychiatr*") OR (AB "perinatal psychiatr*") OR (SU "perinatal psychiatr*") | Expanders - Apply equivalent subjects Search modes - Proximity |  | 1,330 | [Edit](javascript:__doPostBack('ctl00$ctl00$MainContentArea$MainContentArea$editControl$printHistory$HistoryRepeater$ctl12$linkEditSearch',''))S11 |
| S10 | (TI "post traumatic stress disorder") or (AB "post traumatic stress disorder") OR (SU "post traumatic stress disorder") or (TI "ptsd") OR (AB "ptsd") OR (SU "ptsd") | Expanders - Apply equivalent subjects Search modes - Proximity |  | 17,348 | [Edit](javascript:__doPostBack('ctl00$ctl00$MainContentArea$MainContentArea$editControl$printHistory$HistoryRepeater$ctl13$linkEditSearch',''))S10 |
| S9 | (TI "delirium") OR (AB "delirium") OR (SU "delirium") OR (TI "neuropsych*") OR (AB "neuropsych*") OR (SU "neuropsych*") | Expanders - Apply equivalent subjects Search modes - Proximity |  | 69,376 | [Edit](javascript:__doPostBack('ctl00$ctl00$MainContentArea$MainContentArea$editControl$printHistory$HistoryRepeater$ctl14$linkEditSearch',''))S9 |
| S8 | (TI "psychogeriatric*") OR (AB "psychogeriatric*") OR (SU "psychogeriatric*") OR (TI "geriatric psychiat*") OR (AB "geriatric psychiat*") OR (SU "geriatric psychiat*") OR (TI "dementia") OR (AB "dementia") OR (SU "dementia") OR (TI "alzheimer*") OR (AB "alzheimer*") OR (SU "alzheimer*") | Expanders - Apply equivalent subjects Search modes - Proximity |  | 115,670 | [Edit](javascript:__doPostBack('ctl00$ctl00$MainContentArea$MainContentArea$editControl$printHistory$HistoryRepeater$ctl15$linkEditSearch',''))S8 |
| S7 | (TI "child adolescent psychiatr*") OR (AB "child adolescent psychiatr*") OR (SU "child adolescent psychiatr*") OR (TI "child/adolescent psychiatric assess*") OR (AB "child/adolescent psychiatric assess*") OR (SU "child/adolescent psychiatric assess*") | Expanders - Apply equivalent subjects Search modes - Proximity |  | 161 | [Edit](javascript:__doPostBack('ctl00$ctl00$MainContentArea$MainContentArea$editControl$printHistory$HistoryRepeater$ctl16$linkEditSearch',''))S7 |
| S6 | (TI "autism spectrum disorder") OR (AB "autism spectrum disorder") OR (SU "autism spectrum disorder") OR (TI "asd") OR (AB "asd") OR (SU "asd") OR (TI "autis*") OR (AB "autis*") OR (SU "autis*") OR (TI "attention deficit hyperactivity disorder") OR (AB "attention deficit hyperactivity disorder") OR (SU "attention deficit hyperactivity disorder") OR (TI "adhd") OR (AB "adhd") OR (SU "adhd") OR (TI "neurodevelopmental disorder*") OR (AB "neurodevelopmental disorder*") OR (SU "neurodevelopmental disorder*") OR (TI "intellectual disabilit*")OR (AB "intellectual disabilit*") OR (SU "intellectual disabilit*") | Expanders - Apply equivalent subjects Search modes - Proximity |  | 94,157 | [Edit](javascript:__doPostBack('ctl00$ctl00$MainContentArea$MainContentArea$editControl$printHistory$HistoryRepeater$ctl17$linkEditSearch',''))S6 |
| S5 | (TI "anxiety disorder*") OR (AB "anxiety disorder*") OR (SU "anxiety disorder*") OR (TI "obsessive-compulsive disorder") or (AB "obsessive-compulsive disorder") OR (SU "obsessive-compulsive disorder") OR (TI "obsessive compulsive disorder") OR (AB "obsessive compulsive disorder") OR (SU "obsessive compulsive disorder") OR (TI "ocd") OR (AB "ocd") OR (SU "ocd") | Expanders - Apply equivalent subjects Search modes - Proximity |  | 28,559 | [Edit](javascript:__doPostBack('ctl00$ctl00$MainContentArea$MainContentArea$editControl$printHistory$HistoryRepeater$ctl18$linkEditSearch',''))S5 |
| S4 | (TI "psychotherap*") OR (AB "psychotherap*") OR (SU "psychotherap*") OR (TI "therap*") OR (AB "therap*")(SU "therap*") OR (TI "counsel*") OR (AB "counsel*")(SU "counsel*") OR (TI "therapist*") OR (AB "therapist*") OR (SU "therapist*") OR (TI "cognitive behavio* therap*") OR (AB "cognitive behavio* therap*") OR (SU "cognitive behavio* therap*") | Expanders - Apply equivalent subjects Search modes - Proximity |  | 381,818 | [Edit](javascript:__doPostBack('ctl00$ctl00$MainContentArea$MainContentArea$editControl$printHistory$HistoryRepeater$ctl19$linkEditSearch',''))S4 |
| S3 | (TI "major depress* disorder*") OR (AB "major depress* disorder*") OR (SU "major depress* disorder*") OR (TI "mdd") OR (AB "mdd") OR (SU "mdd") OR (TI "major depression") OR (AB "major depression") OR (SU "major depression") OR (TI "bipolar disorder") OR (AB "bipolar disorder") OR (SU "bipolar disorder") OR (TI "affective disorder*") OR (AB "affective disorder*") OR (SU "affective disorder*") OR (TI "mania") OR (AB "mania") OR (SU "mania") OR (TI "manic") OR (AB "manic") OR (SU "manic") | Expanders - Apply equivalent subjects Search modes - Proximity |  | 43,776 | [Edit](javascript:__doPostBack('ctl00$ctl00$MainContentArea$MainContentArea$editControl$printHistory$HistoryRepeater$ctl20$linkEditSearch',''))S3 |
| S2 | (TI "psychosis") OR (AB "psychosis")(SU "psychosis") OR (TI "psychotic") OR (AB "psychotic") OR (SU "psychotic") OR (TI "schizophreni*") OR (ab "schizophreni*") OR (SU "schizophreni*") | Expanders - Apply equivalent subjects Search modes - Proximity |  | 51,394 | [Edit](javascript:__doPostBack('ctl00$ctl00$MainContentArea$MainContentArea$editControl$printHistory$HistoryRepeater$ctl21$linkEditSearch',''))S2 |
| S1 | (TI "psychiatr*") OR (AB "psychiatr*") OR (SU "psychiatr*") OR (TI "mental health") OR (AB "mental health") OR (SU "mental health") OR (TI "mental illness") OR (AB "mental illness") OR (SU "mental illness") or (TI "mental disorder*") or (AB "mental disorder*") OR (SU "mental disorder*") OR (TI "psychiatric illness*") OR (AB "psychiatric illness*") OR (SU "psychiatric illness*") OR ( mental* PRE/3 ( patient* OR inpatient* OR outpatient* OR client* OR consumer* ) ) ) OR ( ( psychiat* PRE/3 ( patient* OR inpatient* OR outpatient* OR client* OR consumer* ) ) ) | Expanders - Apply equivalent subjects Search modes - Proximity |  |  |  |
