# Supplementary material for: Effects of face coverings on people and interactions in mental health settings: scoping review – ERRATUM
Source: BJPsych Open. 2026 Jan 7;12(1):e32. doi: 10.1192/bjo.2025.10962 (PMC12835721; doi:10.1192/bjo.2025.10962)
Supplement: Van Houtte et al. supplementary material [file S2056472425109629sup003.docx]

| Author & Year | Country | Aims | Population and sample size | Methodology | Relevant key findings |
| --- | --- | --- | --- | --- | --- |
| Bani et al., 2023 [(30)](https://www.zotero.org/google-docs/?W8gpK0) | Italy | Assess the impact of masks on clinicians’ perception of clinical interactions and ability to read facial expressions. | n = 342  Psychologists and psychotherapists | Survey and emotion-recognition task. | Masks impaired the self‐perceived ability of clinicians to build effective relationships and to communicate effectively with patients, and reduced satisfaction in clinical encounters. The ability of clinicians to recognize facial emotions was reduced for masked happy and angry faces. |
| [Biermann et al., 2022](https://www.zotero.org/google-docs/?fq1xi7)  [(34)](https://www.zotero.org/google-docs/?bm0jUN) | Germany | Explore how trustworthiness and happiness judgements in borderline personality disorder are affected by facemasks. | n = 149  Adult females in the community  67 with borderline personality disorder 75 controls | Survey and emotion-recognition task | The borderline personality disorder group had overall lower happiness and trustworthiness perceptions and lower confidence in their judgments than controls, when interpreting emotions for both masked and non-masked faces. Masked faces were judged as less happy and less trustworthy, and they reduced confidence in these social judgements for all participants, with both groups equally impacted. |
| [Clegg et al., 2024](https://www.zotero.org/google-docs/?GowVZt)  [(48)](https://www.zotero.org/google-docs/?wH4ySP) | UK | Explore autistic people’s experiences of wearing face masks and interacting with other people who are wearing face masks. | n = 49  Adults with ASD (73% with formal diagnoses) | Survey | Sensory issues when wearing masks were a common challenge (65%). The majority of participants reported negative impacts of masks on social interactions (51%) including difficulties understanding or being understood due to loss of lip reading and facial expression cues. Some participants (20%) also reported benefits of face masks on social interactions including less pressure to “camouflage” their own facial expressions or to recognise others. |
| [Doan et al., 2021](https://www.zotero.org/google-docs/?Rn7U97)  [(66)](https://www.zotero.org/google-docs/?eS6BYU) | UK | Explore burnout rates among psychiatrists during the COVID-19 pandemic | n = 106 Psychiatrists and trainees | Survey | Face masks were felt to affect rapport with patients by 62% of participants. |
| [Dondé et al., 2022](https://www.zotero.org/google-docs/?IVsRgt)  [(45)](https://www.zotero.org/google-docs/?msGVEZ) | France | Explore opinions and attitudes of psychiatrists on the effects of face masks on the psychiatric consult. | n = 513  Psychiatrists and trainees | Survey | Participants reported that when clinicians and / or patients were wearing masks, it was harder to collect clinical signs/symptoms from both verbal and non-verbal cues (61-78%), the quality of practice deteriorated (94%), false inferences or beliefs were more common (78-89%), and patient-clinician interactions were worse (72–75%). A negative impact on the therapeutic alliance was reported by 47% of participants. The perceived impact was comparable whether the patient or the psychiatrist was wearing a mask. |
| [Eddy, 2021](https://www.zotero.org/google-docs/?dvijvj)  [(27)](https://www.zotero.org/google-docs/?lndt2B) | UK | Explore the perceptions of employees of a mental health trust on the social impacts of the COVID‐19 pandemic. | n = 464  Employees of a mental health trust (56% working in clinical roles) | Survey | Most participants thought face masks could negatively impact interactions with service users (65%) and colleagues (62%). Most participants thought masks made it more difficult to communicate with service users (77%) and colleagues (66%). Themes arising from feedback comments included that masks were a distraction, caused discomfort and a deterioration in clinician morale. Most (62%) believed that the benefits of face masks outweighed the costs. |
| [Erschens et al., 2022](https://www.zotero.org/google-docs/?GJQizO)  [(40)](https://www.zotero.org/google-docs/?SV77DH) | Germany | Explore the experience of wearing masks on psychotherapy in an inpatient setting | n = 95  62 adult inpatients  33 clinicians practicing psychotherapy | Survey | Clinicians reported negative impacts of masks on forming relationships (86%) experiencing connectedness with colleagues (65%), and on their own wellbeing (54%), and caused feelings of alienation / anonymization (45%), denying they created a “We-feeling” (85%). Clinicians felt masks hindered therapeutic treatment (65-85%), and caused difficulties recognising the mood state. Clinicians were more likely to report these negative associations than patients. Masks were reported to interfere with therapy by 11 - 45% of patients (depending on therapy type). The majority of patients rated their psychotherapy as highly profitable despite masks. Some patients reported that masks inhibit personal contact, caused misinterpretation, misunderstanding and problems perceiving emotions, as well as feelings of increased anxiety, panic and breathing difficulties. |
| [Escelsior et al., 2022](https://www.zotero.org/google-docs/?V5zfo5)  [(26)](https://www.zotero.org/google-docs/?5D3QYT) | Italy | Explore the impact of masks on emotion recognition for psychiatric patients | n = 73  45 adult psychiatric inpatients (diagnoses: bipolar disorder n=13, major depression n=19, schizophrenia n=13)  28 healthy controls | Emotion-recognition task | Covering faces with masks reduced emotion recognition (ER) accuracy in most participants. The largest negative impact of masks on ER was observed when participants with depression or schizophrenia had to identify emotions with positive valence at a low-intensity level. Participants with bipolar disorder had similar performances to controls. |
| [Gehdu et al., 2023](https://www.zotero.org/google-docs/?g1OB0N)  [(35)](https://www.zotero.org/google-docs/?fNTsgo) | UK | Investigate whether mask-related emotion-recognition deficits for autistic people is related to diagnosis or alexithymia | n = 132  66 autistic adult patients  66 non-autistic controls | Emotion recognition task | ER accuracy was lower for autistic participants overall compared to non-autistic controls when evaluating both masked and unmasked faces, with a similar drop in performance for both groups with masks present. When separated into subgroups, those autistic participants with low levels of alexithymia scored similarly to controls for both masked and unmasked faces. |
| [Imai and Furukawa, 2021](https://www.zotero.org/google-docs/?Y4Dzsa)  [(29)](https://www.zotero.org/google-docs/?OSLGH6) | Japan | Explore the effects of masks on communication and fear of infection in psychiatric patients | n = 425  Adult outpatients (varied diagnoses including neurotic, stress-related, somatoform, mood disorders); first-visit patients excluded | Survey | The majority of patients (91%) reported no change in “difficulty describing [their] emotions and thoughts to the doctor compared to before the doctor started wearing a mask/using a plastic partition”. The majority of patients reported a reduction (53%) or no change (34%) in anxiety about infection risk “compared to before the doctor started wearing a mask/using a plastic partition”. |
| [Kidd, 2023](https://www.zotero.org/google-docs/?XM8Rtu)  [(41)](https://www.zotero.org/google-docs/?UuGYUu) | England | Explore impact of masks on relational depth in therapy | n = 4  Female psychotherapists | Semi-structured interviews | Four themes emerged through interpretive phenomenological analysis:  1. Constant visual reminder of pandemic: masks as an intrusive presence and barriers to autonomous practice due to mandates  2. Loss: of self/therapeutic identity, visual cues, quality of connection, internal signals.  3. Masked disinhibition: Maskenfreiheit or freedom in conjunction with anonymity and disconnection.  4. Potential for “depth through disconnection”.  Participants also noted that attitudes towards masks are mediated by “variable symbolic meaning of masks”. |
| [Kuczyk et al., 2024](https://www.zotero.org/google-docs/?ltFPBt)  [(28)](https://www.zotero.org/google-docs/?NZRLsA) | Germany | Compare the expectations of patients regarding wearing a mask in psychotherapy before the start of therapy with the final experience after the end of therapy. | n = 142  Adults,inpatients and outpatients (varied diagnoses including major depression (93%), anxiety disorders, OCD, PTSD, personality disorder, somatoform disorder, eating disorder. | Survey | Patients perceived significantly fewer communication barriers with face masks than they had first expected.  Patients’ attitudes towards masks correlated with their expectations and experiences. Patients who reported having more “negative attitudes” towards masks were more likely to experience negative aspects of mask-wearing in psychotherapy. |
| [Kuusikko-](https://www.zotero.org/google-docs/?fIULUt)  [Gauffin et al., 2018](https://www.zotero.org/google-docs/?H03PHg)  [(25)](https://www.zotero.org/google-docs/?j7vscv) | Finland, Egypt | Examine emotion recognition patterns using the eye region, comparing children with and without ASD | n = 128  66 children with ASD  62 typically developing children | Emotion recognition task. | Children with ASD had deficits in emotion recognition from the eye region compared to typically developing peers. Some differences were found between the two cultures studied. |
| [Lau et al., 2022](https://www.zotero.org/google-docs/?zIeXaj)  [(42)](https://www.zotero.org/google-docs/?5h2BbX) | Germany | Investigate how people with pre-existing mental health conditions and healthcare profession-  als experienced changes linked to the COVID-19 pandemic. | n=32  19 adult inpatients (varied diagnoses including mood, anxiety, eating, somatoform, personality disorders, PTSD and ASD)  13 clinicians (psychologists, psychotherapists, nurses) | Semi-structured interviews | Masks were associated with reduced freedom of expression, feelings of alienation, discomfort, altered self-perception and perception of others, communication impairments, reduced facial recognition, hindered therapeutic relationship, reduction of interpersonal synchrony, and triggering insecurities in psychotherapy. |
| [Moosavi et al., 2024](https://www.zotero.org/google-docs/?eqtM92)  [(31)](https://www.zotero.org/google-docs/?eP2pHj) | Germany | Explore the impact of masks on emotion recognition for females with major depression. | n = 61  31 adult female inpatients with major depression  30 female controls | Emotion-recognition tasks. | Masks disproportionately affected emotion recognition in females with major depression compared with non-depressed controls. Between the two tests used, there was variation in which emotions were more difficult to accurately identify. |
| [Murphy et al., 2022](https://www.zotero.org/google-docs/?Ej2Cti)  [(49)](https://www.zotero.org/google-docs/?fpPsaU) | Canada | Explore experiences of pharmacists providing services to people with lived experience of mental illness. | n = 60  Pharmacists and technicians/assistants | Survey | The majority (53%) of participants agreed that their ability to perform their role in the pharmacy was negatively affected by patients’ use of masks. |
| [Okyar Baş et al., 2022](https://www.zotero.org/google-docs/?P08rFz)  [(43)](https://www.zotero.org/google-docs/?5YH7U5) | Turkey | Evaluate the possible effects of face masks on the cognitive test performance of older adults. | n=198  37 outpatients with Alzheimer’s disease  30 with mild cognitive impairment  129 controls | Observational study  Cognitive screening tests | Mask use may reduce diagnostic accuracy of cognitive screening tests, which could lead to overdiagnosis. |
| [Ribeiro et al., 2021](https://www.zotero.org/google-docs/?6JXgZl)  [(38)](https://www.zotero.org/google-docs/?rpYRZW) | Portugal | Explore how psychotherapists understand the therapeutic process using a face mask, comparing existing with new clients. | n= 137  Psychotherapists | Survey | From the quantitative data (Likert scale), face masks had no major perceived impact on therapy quality dimensions.  From the qualitative data (free text), the majority of therapists found working with masks to have been a demanding experience, with difficulties in communication (verbal, non-verbal, reading facial expression) for both new and existing clients. Between 20-50% therapists found difficulties in therapeutic work collecting information, reading/expressing emotions (new and existing clients), difficulties building rapport/emotional bonding (new clients) |
| [Schnitzler et al., 2024](https://www.zotero.org/google-docs/?GACWdS)  [(36)](https://www.zotero.org/google-docs/?Sg0RX8) | Germany | Assess whether emotion recognition in ASD is affected by partial face covering | n = 72  36 adults with ASD  36 controls | Emotion recognition task | Emotion recognition accuracy was lower for covered faces than uncovered faces for both groups. People with ASD were less accurate at reading emotions than controls for both covered and uncovered faces, with a proportionate loss of emotion recognition accuracy when face masks were present. |
| [Silva et al., 2023](https://www.zotero.org/google-docs/?SbILgz)  [(50)](https://www.zotero.org/google-docs/?RdMPNa) | Brazil | Explore the perceptions of nurses on the repercussions of the COVID-19 pandemic on care for children with mental disorders in a pediatric inpatient unit. | n=13  Nurses working at paediatric inpatient unit | Semi-structured interviews | Nurses felt the mandatory use of PPE (specifically masks) impacted delivery of care, interfered with the physical and emotional approach especially for those with schizophrenia or ASD, frightened some children and worsened speech difficulties. |
| [Tamon et al., 2022](https://www.zotero.org/google-docs/?PPx5yt)  [(75)](https://www.zotero.org/google-docs/?2FN5iD) | Japan | Examine relationship between ‘restricted interest and repetitive behaviour’ (RRB) characteristics and impact of masks on social communication for children and adolescents with ASD | n=102  Caregivers of children and adolescents with ASD | Survey | Children and adolescents with ASD had difficulties with recognising others’ emotions while wearing masks.  Higher frequencies of pre-pandemic RRB among participants was associated with more difficulties wearing masks. Lower-order RRBs (such as sensory seeking, repetitive motor mannerisms/movements, rituals and routines) were associated with more difficulty with social communication when masked. |
| [Tate et al., 2024](https://www.zotero.org/google-docs/?5WfEhI)  [(32)](https://www.zotero.org/google-docs/?Mqsd6C) | USA | Investigate impacts of face masks on emotion recognition for students with autism | n=14  Students with ASD  (unspecified ages, 3 - 21) No control population | Emotion recognition task | Students with ASD showed an impaired ability to emotionally appraise masked faces for happy and sad emotions, but not anger. |
| [Terkildsen et al., 2024](https://www.zotero.org/google-docs/?C0VC66)  [(39)](https://www.zotero.org/google-docs/?rFWWwv) | Denmark | Explore perspectives of forensic psychiatric patients on COVID-19 prevention measures | n=11  Adult inpatients (diagnoses not recorded) | Semi-structured interviews | Some impacts of masks included relational barriers with staff (for newer patients), difficulties reading facial expression, and increasing misunderstandings.  Masks did not have a significant effect on relations for those admitted prior to introduction of masks. |
| [Thomas and Tranel, 2023](https://www.zotero.org/google-docs/?B1EWUp)  [(44)](https://www.zotero.org/google-docs/?bbMeQ6) | USA | Investigate effects of mask-wearing on adult neuropsychological test performance | n=1591 Adults with cognitive disorders at neuropsychiatric clinic  837 masked  754 pre-pandemic / unmasked | Retrospective observational study | Mask-wearing (by both patient and examiner) during neuropsychological assessment compromised performance on verbally mediated tests, but not visually-mediated tests in older patients. |
| [Tso et al., 2022](https://www.zotero.org/google-docs/?1eSlQ8)  [(37)](https://www.zotero.org/google-docs/?zfP0Q6) | China | Investigate effect of masks on face learning and recognition in adults with autism | n=61  29 adults with ASD  32 controls | Face recognition task | When faces were first learned unmasked, both autistic and non-autistic participants experienced similar drops in their facial recognition performance when the faces were then masked, with autistic participants having a lower baseline.  When faces were first learned with a mask, autistic participants performed significantly worse with facial recognition when masks were removed, compared with non-autistic controls. |
| [Ventura et al., 2023](https://www.zotero.org/google-docs/?uKdGaz)  [(33)](https://www.zotero.org/google-docs/?wI8I1y) | Italy | Investigate the impact of masks on facial recognition and emotion recognition in adults with autism. | n=158  48 adults with ASD  110 controls | Face recognition and emotion recognition tasks | There was an overall disruptive effect of facemasks on identity and emotion recognition for both ASD and controls. Autistic individuals generally had lower levels of performance than controls, and were disproportionately impacted by masks in identity recognition when learning faces with masks, as well as in emotion recognition for fear, happiness and sadness. |
| [Wyler et al., 2021](https://www.zotero.org/google-docs/?hFhllM)  [(46)](https://www.zotero.org/google-docs/?P62O1m) | Switzerland | Explore how adults with ADHD and their therapists experienced therapy during the pandemic in 3 different settings: face-to-face with masks, via telephone, or videoconferencing. | n=163  66 adults with ADHD  97 therapists | Survey | A majority of therapists felt masks caused verbal and non-verbal communication difficulties. A pre-existing bond of trust lessened the negative impact of masks.  From the patient perspective, a minority of patients (17%) actively commented on the negative impacts of masks on communication on open questioning about their therapy. In general patients preferred not having masks. |
| [Zhang et al., 2024](https://www.zotero.org/google-docs/?KKJsEM)  [(47)](https://www.zotero.org/google-docs/?OwN3i7) | China | Explore the effect of surgical masks and n95s on patients with anxiety disorders | n= 90  30 controls (no mask)  30 surgical mask  30 N95  All participants were adult outpatients with first episode anxiety disorders | Randomized controlled trial | Prolonged mask use significantly exacerbated anxiety symptoms. This impact was larger with N95 use compared with surgical mask use. |
